# Five-Decade Update on Chemopreventive and Other Pharmacological Potential of Kurarinone: a Natural Flavanone

**DOI:** 10.3389/fphar.2021.737137

**Published:** 2021-09-27

**Authors:** Shashank Kumar, Kumari Sunita Prajapati, Mohd Shuaib, Prem Prakash Kushwaha, Hardeep Singh Tuli, Atul Kumar Singh

**Affiliations:** ^1^ Molecular Signaling & Drug Discovery Laboratory, Department of Biochemistry, Central University of Punjab, Bathinda, India; ^2^ Department of Biotechnology, Maharishi Markandeshwar (Deemed to be University), Ambala, India

**Keywords:** kurarinone, flavanone, anticancer, apoptosis, migration, pharmacological activity

## Abstract

In the present article we present an update on the role of chemoprevention and other pharmacological activities reported on kurarinone, a natural flavanone (from 1970 to 2021). To the best of our knowledge this is the first and exhaustive review of kurarinone. The literature was obtained from different search engine platforms including PubMed. Kurarinone possesses anticancer potential against cervical, lung (non-small and small), hepatic, esophageal, breast, gastric, cervical, and prostate cancer cells. *In vivo* anticancer potential of kurarinone has been extensively studied in lungs (non-small and small) using experimental xenograft models. In *in vitro* anticancer studies, kurarinone showed IC_50_ in the range of 2–62 µM while *in vivo* efficacy was studied in the range of 20–500 mg/kg body weight of the experimental organism. The phytochemical showed higher selectivity toward cancer cells in comparison to respective normal cells. kurarinone inhibits cell cycle progression in G2/M and Sub-G1 phase in a cancer-specific context. It induces apoptosis in cancer cells by modulating molecular players involved in apoptosis/anti-apoptotic processes such as NF-κB, caspase 3/8/9/12, Bcl2, Bcl-XL, etc. The phytochemical inhibits metastasis in cancer cells by modulating the protein expression of Vimentin, N-cadherin, E-cadherin, MMP2, MMP3, and MMP9. It produces a cytostatic effect by modulating p21, p27, Cyclin D1, and Cyclin A proteins in cancer cells. Kurarinone possesses stress-mediated anticancer activity and modulates STAT3 and Akt pathways. Besides, the literature showed that kurarinone possesses anti-inflammatory, anti-drug resistance, anti-microbial (fungal, yeast, bacteria, and Coronavirus), channel and transporter modulation, neuroprotection, and estrogenic activities as well as tyrosinase/diacylglycerol acyltransferase/glucosidase/aldose reductase/human carboxylesterases 2 inhibitory potential. Kurarinone also showed therapeutic potential in the clinical study. Further, we also discussed the isolation, bioavailability, metabolism, and toxicity of Kurarinone in experimental models.

## Introduction

Plant-based secondary metabolites are indirectly helpful for the growth and development of the plants, but it is of direct importance for humans. The plant-based phytochemical/secondary metabolites can be categorized in various groups majorly based on their structural skeleton and functional groups (Kumar, 2014). Our research group reported the pharmaceutical activities of different plants and/or phytochemicals (Kumar and Pandey 2013; Mishra et al., 2013; Kumar et al., 2014; Kushwaha et al., 2019a; Kushwaha et al., 2020a; Kushwaha et al., 2020b; Kushwaha et al., 2021). Flavonoids are a group of polyphenolic compounds that occur widely in plants. They contain a benzo-γ-pyrone structure comprised of two benzene rings (A and B), connected with a heterocyclic pyran ring (C). Flavonoids are classified as flavone, flavanol, flavanone, and others. These groups may differ from each other based on the oxidation level and type of substitution group at the C ring. They are well known for their pharmacological potential such as anticancer, antioxidant, neuroprotective, hepatoprotective, immune-modulatory, antimicrobial, antidiabetic, etc (Kushwaha et al., 2019b). Kurarinone is a natural flavanone found in different plants and possesses various pharmacological activities including chemoprevention efficacy. Literature showed anticancer, anti-fungal, anti-bacterial, anti-Corona virus, neuroprotective, anti-drug resistance, antioxidant, and anti-inflammatory potential of kurarinone. In the present review, we discuss the chemoprevention potential of kurarinone and the underlying regulatory mechanism in detail. The various pharmacological activities such as Ca^+^ channel and glucose transporter activity modulation, metabolic enzyme, and xenobiotic metabolism enzyme inhibition potential are reviewed. The bioavailability and toxicity of a therapeutic agent are of prime concern. Thus, we also reviewed the studies based on these parameters of kurarinone.

### Kurarinone Chemistry and Natural Sources

Kurarinone is a naturally occurring prenylated flavanone. Komatsu et al. (1970) for the first time isolated kurarinone from the methanolic root extract of *Sophora angustifolia* (Fabaceae). Briefly, the extract was chromatographed and eluted using acetone:hexane (1:1 ratio). The eluted material was subjected to thin-layer chromatography and the relatively slower-moving fraction was taken and re-chromatographed using chloroform:methanol (95:5 ratio) as elution solvent which yielded 15 g kurarinone (started with 20 kg raw plant material). Kurarinone was obtained as a colorless crystalline substance. The structure was established using UV, IR, and NMR techniques (Komatsu et al., 1970). Later, Yamahara et al. (1990) isolated the kurarinone from *Sophora flavescens* root extract, which became a choice of isolation source for the scientist in the two decades. Isolation of kurarinone from different sources and plant parts has been summarized in Table 1. Kurarinone contains a lavandulyl group at the C-8 position and a methoxy group at the C-5 position along with hydroxyl groups at positions C-2, C-4, and C-7. Chung et al. (2004) reported that the lavandulyl group and the positions of the hydroxyl group are important for the diacylglycerol acyltransferase inhibitory activity of kurarinone. Son et al. (2003) reported that the lavandulyl group and methoxy groups of kurarinone play important roles in tyrosinase inhibitory activity.

**TABLE 1:** Kurarinone isolation from different sources.

S. No	Source	Solvent	Part/type	Ref
1	*Sphora flavescens Ait*	Ethyl acetate extract	Root	Yamahara et al. (1990)
2	*Gentiana macrophylla*	Aqueous acetone	Root	Tan et al. (1996)
3	*S. flavescens Ait*	—	Root	Kang et al. (2000)
4	*S. flavescens Ait*	Dichloromethane fraction	—	Kim et al. (2003)
5	*Albizzia julibrissin* (Leguminosae)	EtOAc fraction of the MeOH extract	—	Jung et al. (2004)
6	*Sophora flavescens*	Polyphenolic extract	Root	De Naeyer et al. (2004)
7	*S. flavescens Ait*	MeOH extracts	—	Lee et al. (2005)
8	*S. flavescens Ait*	—	—	Zhang et al. (2007)
9	*S. flavescens*	—	Root	Li et al. (2008)
10	*S. flavescens*	—	—	Ma et al. (2013)
11	*S. tonkinensis*	—	—	He et al. (2013)
12	*S. flavescens*	—	Root	Zhang et al. (2013)
13	*S. flavescens*	—	—	Zhang et al. (2016a)
14	*S. flavescents*	—	Flower	Zhang et al. (2016b)
15	Traditional Chinese Medicine Xin-Su-Ning capsule (XSNC)	—	—	Guo et al. (2018)
16	*S. flavescens*	—	—	Zhou et al. (2018)

### Anticancer Potential

Kang et al. (2000) first reported the anticancer efficacy of *Sophara flavescens*. To isolate the compounds, the methanol extract was prepared and partitioned between dichloromethane and aqueous methanol. Further through re-chromatography kurarinone was isolated in a sub-fraction. The methanol extract of the *S. flavescens* root and isolated compounds showed potential anticancer efficacy against HL-60 cells (human myeloid leukemia). Kurarinone depicted 18.5 µM IC_50_ in comparison to the standard drug cisplatin (2.3 µM IC_50_) (Kang et al., 2000). It has been reported that chemotherapy induces the nuclear factor NF-κB pathway which in turn results in the activation of survival signaling and molecular events involved in anti-apoptosis. By doing this, the NF-κB pathway activation plays an important role in cancer drug resistance. Bcl2, an important NF-κB pathway target gene, is involved in anti-apoptotic events and drug resistance in clinical oncology. Han et al. (2007) studied the efficacy of kurarinone on NF-κB pathway and apoptosis induction. Further, the study explored the effect of test samples on the activity of different receptor tyrosine kinases involved in clinical oncology. The *in vitro* apoptosis induction potential of the kurarinone containing extract was studied in lung and esophageal carcinoma cell lines (H460 and Eca-109, respectively). The *in vivo* apoptosis induction potential of isolated kurarinone alone and in combination with Taxol (standard anticancer drug) was studied in the lung cancer cell line xenograft model. At a dose of 100 mg/kg body weight per day, kurarinone decreased the expression of Bcl2 protein and up regulated the levels of caspase 8 and 3 in the experimental rat model. Kurarinone showed dose-dependent (5.8 µg/ml IC_50_) inhibitory potential on NF-κB pathway activation in lipopolysaccharide induced pathway activation in an experimental model. Kurarinone inhibited induced IκBα phosphorylation (regulates NF-κB nuclear translocation) in HEK293 (kidney) cells at 100 µg/ml concentration. Kurarinone inhibited EGFR and Her-2 phosphorylation (in A431 and MDA-MB-453 cell lines) at 20 µg/ml concentration. The EGFR activity was also inhibited in *in vivo* experimental rats at 100 and 500 mg/kg body weight treatment with 14 and 40% inhibition efficacy, respectively. Moreover, kurarinone showed inhibition of KDR activity with 2.3 µM IC_50_ (Han et al., 2007).

**FIGURE 1:**
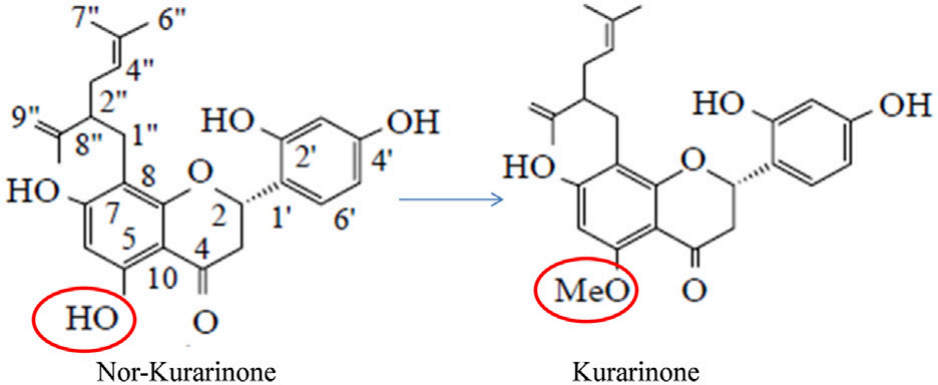
Structural difference between nor-kurarinone and kurarinone.

Sun et al. (2008) studied the anti-tumor efficacy of *S. flavescens* flavonoids in *in vitro* (A549, SPC-A-1, NCI-H460 cell lines) and *in vivo* (lung xenograft model) cancer models. The study showed that the phytochemical did not produce toxicity in the experimental rats up to 750 mg/kg bodyweight treatment. The flavonoids showed significant anti-tumor potential in lung cancer in *in vitro* and *in vivo* models at test concentrations/treatment. The study concluded that *S. flavescens* flavonoids such as kurarinone may be developed as novel anti-tumor candidates (Sun et al., 2008). Berghe et al. (2011) studied the mechanistic aspect of TNF-α induced NF-κB pathway activation in fibroblast L929sA cells. Kurarinone inhibited TNF-α induced IL-6 mRNA expression in transfected L929sA cells significantly at 4 and 40 µM concentrations. Further, the TNF-α induced promoter activity of different genes (IL6, IL8, E-sel, PGK. and NF-κB) were decreased in the presence of kurarinone at 10, 25, 50, and 100 µg/ml concentrations. Kurarinone did not affect the TNF-induced NF-κB binding to DNA but it significantly inhibits p42/p44 ERK phosphorylation thereby inhibiting the downstream effector molecules (p90RSK and target S6RP) at test concentrations. Further, the toxicity of kurarinone was studied in low and high metastatic *in vitro* breast cancer models (MCF7/6 and MDA-MB-231 cells, respectively). Dose-dependent activity was observed in the test cells (Berghe et al., 2011). In an interesting study, Shi et al. (2012a) studied the microbiologically transformed nor-kurarinone compounds for their anticancer potential. A total of seven compounds (kurarinone was one of them) was formed by the action of *Cunninghamella blakesleana* on the test compound and the structure was confirmed using NMR and MS techniques. Kurarinone showed toxicity against Hela and A375 cell lines with an IC_50_ of 36 and 62 µM, respectively (Shi et al., 2012a) (Figure 1). The same research group obtained some new glycosylated compounds by transforming kurarinone in the presence of *Cunninghamella* spp. One of the transformed products named kurarinone-7-O-β-glucoside showed 8.7 µmol/IC_50_ against Hela cells (Shi et al., 2012b).

Seo et al. (2012) reported the effect of kurarinone on TRAIL (TNF-related apoptosis inducing ligand) induced apoptosis and associated mechanism in Hela cells. Results showed that kurarinone exerts apoptosis induction potential in a caspase-dependent manner at 5 µM concentration. It showed effect neither on Bcl2 and inhibitor of apoptosis (IAP) family proteins nor on death receptors (DR4 and 5) induced cytotoxicity. TRAIL is known to induce apoptosis through TRAIL-R1/R2 (DR4/5) transmembrane receptors. Further results showed that kurarinone has the ability to potentiate the apoptosis induction potential of TRAIL via inhibiting the NF-κB mediated cFLIP (FLICE-inhibitory protein long form) expression (Seo et al., 2012). TRAIL is an important anticancer agent, which induces apoptosis in different types of cancer cells. Gastric cells are known to be less sensitive to TRAIL induced apoptosis. Zhou et al. (2015) studied the effect of kurarinone and TRAIL co-treatment in gastric cells (SGC7901). Kurarinone showed significant cytotoxicity to gastric cells at 10 µM or higher concentrations. Co-treatment with the TRAIL (50 ng/ml), kurarinone showed toxicity even at lower concentrations (5 µM). Similarly, the co-treatment significantly increased the cleaved caspase-3 and PARP proteins in comparison to treatment alone. Further, the co-treatment arrested the gastric cells in the G2/M phase and decreased the cyclin B1 and cyclin A protein expression. Kurarinone-TRAIL treatment decreased the apoptotic regulator proteins (Mcl-1 and c-FLIP) at mRNA and protein level. The result showed that the co-treatment decreases the phosphorylation of STAT3 protein which is required for the expression of Mcl-1 and c-FLIP proteins (Zhou et al., 2015).

Recently, Yang et al. (2018) studied in detail the anticancer efficacy of kurarinone in non-small cell lung cancer (NSCLC) *in vitro* and *in vivo* models. The author proposed the underlying apoptosis induction mechanism of kurarinone in NSCLC cells (A549). Kurarinone showed little toxicity on normal human bronchial epithelial cells (BEAS-2B) at 5–25 µM concentration. The phytochemical produced dose-dependent apoptosis induction potential by decreasing the Bcl2-Bax protein ratio, activating caspase 9/3, decreasing Grp78 expression, inhibiting caspase 12/7, and suppressing the Akt activity at 5, 10, and 25 µM concentrations. Kurarinone did not produce toxicity in experimental rats and produced anti-cancer efficacy in the A549 xenograft rat model at a dose of 20 and 40 mg/kg body weight. Kurarinone treatment significantly reduced the tumor weight and volume in comparison to the non-treated group in 27 days of treatment (Yang et al., 2018). In a different study, Chung et al. (2019) studied the anticancer effect of kurarinone in small-cell lung cancer (SCLC) cells (H1688 and H146) and deduced the underlying mechanism. Kurarinone showed 12.5 and 30.4 µM IC_50_ for H1688 and H146 cancer cell lines, respectively. Early and late apoptotic cell population was increased in kurarinone treated cells. The cleaved PARP level was increased in kurarinone treated cells at 6.25, 12.5, and 25 µM concentrations. Further, the change in cleaved caspase 3, Bcl-2 and Bcl-XL proteins in the presence of kurarinone revealed mitochondria and receptor mediated apoptosis induction in SCLC cells. Kurarinone increased the sub-G1 population of H1688 cells up to 60% at test concentrations. The phytochemical treatment at 3.125, 6.25, and 12.5 µM concentrations increased the E-cadherin level and decreased the vimentin, N-cadherin, and MMP3/2/9 which indicates the epithelial-mesenchymal transition potential in kurarinone (Chung et al., 2019).

**FIGURE 2:**
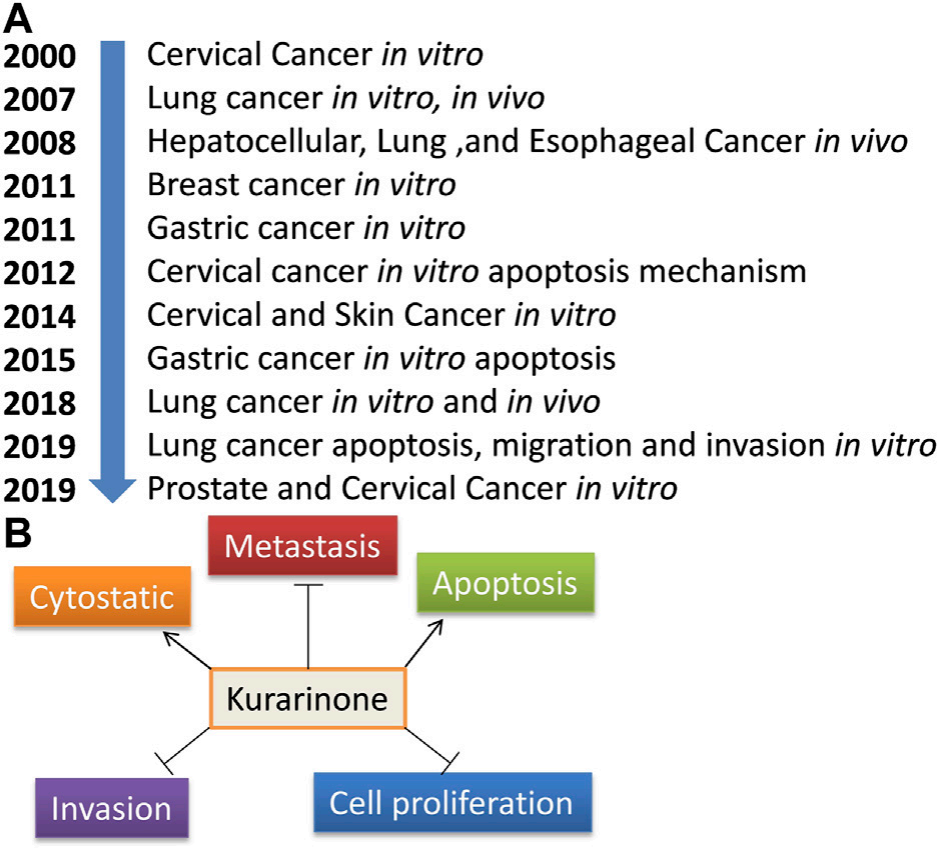
Anticancer studies on kurarinone. **(A)** Year-wise anti-cancer studies on kurarinone, **(B)** cancer hallmarks target by kurarinone.

**FIGURE 3:**
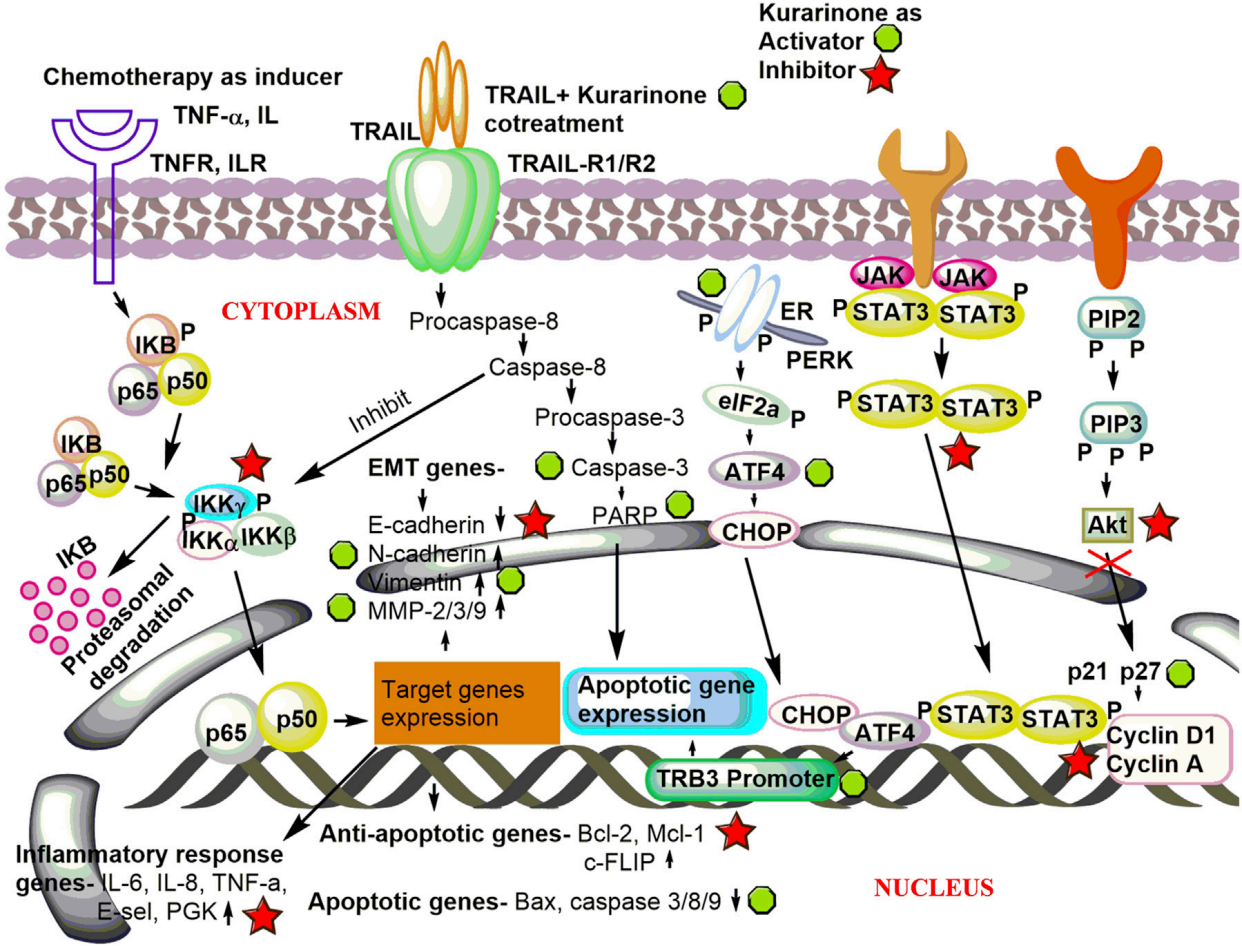
Underlying anticancer mechanism of kurarinone. Kurarinone induces apoptosis and cytostatic effects in cancer cells and inhibits invasion, metastasis, and cellular proliferation. TNF-α, tumor necrosis factor-α; TNFR, tumor necrosis factor receptor; IL, interleukin; ILR, interleukin receptor; TRAIL, TNF-related apoptosis inducing ligand; TRAIL-R1/R2, TNF-related apoptosis inducing ligand-receptor 1/2; EMT, epithelial mesenchymal transition; MMP, matrix metalloproteinase; PGK, phosphoglycerate kinase; cellular FLICE (FADD-like IL-1β-converting enzyme), inhibitory protein; Mcl-1, myeloid leukemia and chlamydia; PARP, poly (ADP-ribose) polymerase; ER, endoplasmic reticulum; PERK, protein kinase R (PRK) like endoplasmic reticulum; eIF2a, eukaryotic translation initiation factor 2A; ATF4, activating transcriptional factor 4; C/EBP, homologous protein; TRB3, Tribbles homolog 3; JAK-STAT, Janus kinase-signal transducer and activator of transcription; PIP2, phosphatidylinositol 4,5-bisphosphate; PIP3, phosphatidylinositol (3,4,5)-trisphosphate; Akt, protein kinase B.

Activating transcriptional factor 4 (ATF4) is an important protein that senses the various stress in the cell (especially ER stress). After activation, it induces stress-relieving and apoptotic genes. Activation of ATF4 by pharmacological agents is a good strategy to target cancer. Nishikawa et al. (2019) reported the effect of kurarinone on ATF4 activation and the cytostatic effect of kurarinone in prostate cancer cells. Kurarinone was isolated from the acetone extract of the *S. flavescens*. A dose dependent anticancer activity was observed in a prostate cancer cell line (PC3) at 10–50 µM concentration (IC_50_ 24.7 µM). A 2.02 selectivity index score was obtained by analyzing the cytotoxic activity of kurarinone in PC3 and normal human diploid fibroblast (TIG3 cells). The index showed the high selectivity of kurarinone toward cancer cells. It showed ATF4 activation and increased expression of its downstream effectors TRB3 in a time (6 and 10 h) and dose (20 and 50 µM) dependent manner at the protein level in prostate cancer cell line (PC3). Kurarinone also increased the TRB3 promoter activity at test concentrations. Kurarinone induced ATF4 activation through the PERK-eIF2α pathway, which was revealed by the activation of PERK by its phosphorylation in the presence of the phytochemical. Further, the cytostatic effect of kurarinone was observed in prostate cancer cells, which was evident by the increased p21 and p27 levels and decrease in cyclin D1 and cyclin A protein expression at 20 and 50 µM concentration (Nishikawa et al., 2019). Recently, Liang et al. (2021) predicted the potential anticancer efficacy of the ingredients of “Compound Kushen Injection” used for lung cancer. The study utilized the network pharmacology approach to find the drug targets, pathway prediction, and protein-protein network analysis for the identified active ingredients including kurarinone (Liang et al., 2021). Pre-clinical anticancer studies on kurarinone from the year 2000 to date show indifferent cancer experimental models, which have been summarized in Figure 2A. The effect of kurarinone on various hallmarks of cancer (Figure 2B) indicates its diversified mode of action in cancer cells. The various anticancer mechanism of kurarinone is depicted in Figure 3.

### Anti-Inflammatory and Immune Response

**FIGURE 4:**
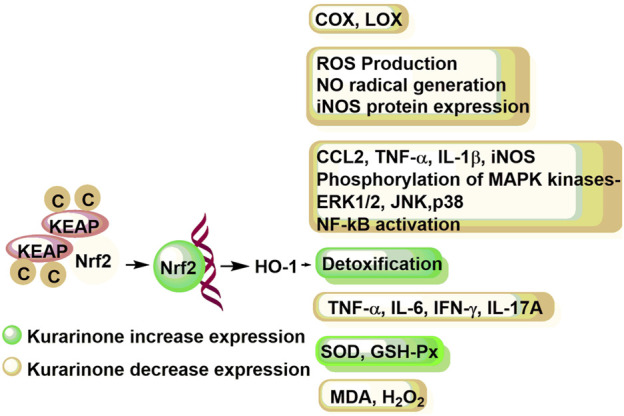
Immune response modulation potential of kurarinone at the molecular level. COX, cyclooxygenase; LOX, lipoxygenase; ROS, reactive oxygen species; NO, nitric oxide; iNOS, inducible nitric oxide synthase; CCL2, C-C motif chemokine ligand 2; TNF-α, tumor necrosis factor-α; IL-1β, interleukin-1β; MAPK, mitogen activated protein kinase; ERL1/2, extracellular signal regulated kinase 1/2; JNK, c-Jun N-terminal kinase; NF-κB, necrosis factor-κB; IL-6, interleukin-6; IL-17A, interleukin-17A; SOD, superoxide dismutase; GSH-Px, plasma glutathione peroxidase; MDA, melanoma differentiation associated protein; H_2_O_2_, hydrogen peroxide; KEAP, Kelch-like-ECH-associated-protein; Nrf2, nuclear factor erythroid 2-related factor 2; HO-1, heme oxygenase-1.

Chi et al. (2001) studied the effect of kurarinone on eicosanoid generating enzymes such as cyclooxygenase 1 and 2 (COX-1 and COX-2) as well as 5- and 12-lipooxygenase (5-LOX and 12-LOX) in bovine platelet and polymorphonuclear leukocytes and monocyte/macrophage cell line (RAW 264.7). Kurarinone inhibited the COX and LOX enzymes in micromolar concentrations. Kurarinone showed better efficacy against the COX-1 enzyme (Chi et al., 2001) (Figure 4). Kim et al. (2002) showed that kurarinone was not able to down-regulate the COX-2 induction in LPS treated RAW cells up to 25 µM concentration. Han et al. (2010) studied the nitric oxide (NO) production, reactive oxygen species (ROS) generation, inflammatory cytokine expression, NF-kB activity, and MAP kinases phosphorylation of kurarinone in lipopolysaccharide (LPS) induced monocyte/macrophage cell line (RAW 264.7). The NO and ROS mediated stress modulates the cytokine production by regulating the NF-kB pathway. The NF-kB translocates to the nucleus and initiates the transcription of target genes responsible for different molecular events (such as stress-response, peptide/cytokine/chemokine secretion, apoptosis inhibitory proteins, etc.). Kurarinone decreased ROS production, NO radical generation, and iNOS protein expression in LPS induced RAW 264.7 cells at micromolar concentration. Similarly, kurarinone reduced the expression of LPS induced expression of inflammatory genes (CCL2, TNF-α, IL-1β, and iNOS) at mRNA level in 24 h treatment. Further, kurarinone decreased the LPS induced phosphorylation of different MAP kinases (ERK1/2, JNK, and p38) in test cell lines and NF-kB activation (Han et al., 2010).

Sahlan et al. (2019) studied the *in vivo* anti-inflammatory potential of propolis from *Tetragronula sp*. and characterized the compounds present in it. The anti-inflammatory potential of the micro-capsulated propolis was studied in the carrageenan-induced rat’s paw inflammation model. LC-MS/MS analysis of the sample revealed that the presence of kurarinone is an active ingredient in propolis. Kurarinone containing propolis produced significant *in vivo* anti-inflammatory potential (Sahlan et al., 2019). Chen et al. (2020) studied the mechanistic efficacy of *S. flavescens* ethanolic extract in dextran sodium sulfate-induced ulcerative colitis rats. Ulcerative colitis (UC) is an immunological disease; if not treated, it may lead to colon cancer. The different pharmacodynamics parameters related to UC were studied. The UHPLC-MS/MS based analysis of the test extract showed the presence of kurarinone in the extract. The study concluded that the compounds such as kurarinone can alter the UT-related pathophysiological conditions (colon length and healthiness of colon tissue, bleeding, etc.) in an experimental rat model (Chen et al., 2020). Kim et al. (2013) studied the immune response inhibitory potential of kurarinone in psoriasis-like skin disease and contact dermatitis experimental chronic inflammatory skin models. The study showed that the phytochemical can decrease the expression of inflammatory molecules (cytokines/interleukins and inflammatory enzymes) and thereby inhibit the JAK/STAT signaling and T-cell receptor pathways. Besides, kurarinone also suppressed the inhibition of the differentiation of CD4(+) T cells in the experimental models. Xie et al. (2018) studied the clinical parameters such as inflammation, demyelination, T helper cells sub-population in sections of the spinal cord, and splenocytes of the multiple sclerosis experimental models. The study reported that kurarinone (100 mg/kg/day) has therapeutic potential against multiple sclerosis mainly by inhibiting Th1 and Th17 cellular proliferation and differentiation.

Nishikawa et al. (2020) studied the anti-inflammatory mode of action of kurarinone *in vitro* using RAW264.7 and HaCaT cells. Kurarinone showed KEAP1 (kelch-like ECH-associated protein 1) down regulation mediated Nrf2 stabilization in a dose dependent manner (at 20–50 µM concentration). Translocation of Nrf2 to the nucleus results in the expression of antioxidant genes (such as heme oxygenase-1 or HO-1) which initiates the detoxification process. Moreover, the study revealed that kurarinone has the potential to inhibit the LPS induced inflammation in RAW264.7 by suppressing the inflammatory mediators through the HO-1 enzyme (Nishikawa et al., 2020). Recently, Tang et al. (2021) studied the effect of kurarinone and its mode of action in collagen induced arthritis mice model. The kurarinone treated experimental group showed decreased serum and paw tissue levels of TNF-α, IL-6, IFN-γ, and IL-17A in collagen-induced arthritis (CIA) mice at 100 mg/kg/day dose. It increased the expression of antioxidant proteins (SOD and GSH-Px) and decreased the MDA production and hydrogen peroxide in paw tissues. Further, the study showed that kurarinone increased the Nrf2 and OH-1 protein expression and decreased the KEAP1 expression in the experimental mice. The study showed that kurarinone exerts the anti-inflammatory action by altering the Th1 and Th17 cell differentiation, increasing antioxidant level and alterations in the Nrf2 pathway in the arthritis animal model (Tang et al., 2021) (Figure 3).

### Effect on Drug Resistance

Choi et al. (1999) tested the anticancer efficacy of the sophora flavanone including kurarinone on P-glycoprotein (Pgp) expressing human colon cancer cell line (HCT15). The effect of kurarinone was also studied on the multi-drug resistance subline of the HCT15 cells (HCT15/CL02). They found that kurarinone showed less activity against HCT15/CL02 cells in comparison to HCT15 cells. Moreover, they reported that combining the treatment with the standard anticancer drug and Pgp inhibitor (Verapamil) also did not increase the cytotoxicity of kurarinone in the test cells. At non-toxic concentrations the kurarinone was not able to increase the paclitaxel mediated cytotoxicity as well as cytoplasmic accumulation of rhodamine 123 dye in the drug resistant cell line. The author concluded that the kurarinoe has no effect on PgP mediated drug resistance in colon cancer cells (Choi et al., 1999). Chen et al. (2005) studied the anti-bacterial efficacy in methicillin and vancomycin antibiotic resistant bacteria *Staphylococcus aureus* (MRSA) and *Vancomycin-resistant enterococci* (VRE), respectively. These two bacteria are among the most common causative agents for lethal hospital infections. They isolated the kurarinone from the roots of *S. flavescens* using HPLC and characterized the compound using NMR spectroscopy. Kurarinone showed 2 µg/ml MIC against both tested drug-resistant bacteria. The author also reported the dose dependent anti-drug resistant potential in kurarinone against MRSA and VRE bacteria (Chen et al., 2005).

### Antimicrobial (Fungal, Yeast, Virus, Bacteria) Activity

Tan et al. (1996) isolated kurarinone from aqueous-acetone root extracts of *Gentiana macrophylla* (Family) using the LC-UV-mass spectrometry method. They reported that kurarinone is active against plant pathogenic fungus and human pathogenic yeast, namely, *Cladosporium cucumerinum* and *Candida albicans*, respectively. The minimum inhibitory concentration (MIC) of kurarinone against the *C. cucumerinum* and *C. albicans* was 5 µg. In the same study, Miconazole and Propiconazole standard growth inhibitors against *C. cucumerinum* and *C. albicans* showed 0.1 and 0.001 µg MIC, respectively (Tan et al., 1996). Sohn et al. (2004) studied the antimicrobial activity of kurarinone (isolated from *S. flavescens* root) against bacteria (*Escherichia coli*, *Salmonella typhimurium*, *Staphylococcus epidermis*, and *S. aureus*) and fungus (*Candida albicans* and *Saccaromyces cerevisiae*) using micro-dilution method-based MIC calculation. Kurarinone showed 60 and 100 µg/ml MIC against all the test fungus and bacteria, respectively. The antibacterial activity was compared with respective standard antibacterial and antifungal compounds such as Ampicillin, Erythromycin, Amphotericin B, Miconazole, and 5-Fluorocytosine (Sohn et al., 2004). Ryu et al. (2008) reported antibacterial activity of kurarinone against Gram-positive/negative bacteria using the paper-disk diffusion method. The *Bacillus subtilis*, *Bacillus cereus*, *Staphylococcus aureus*, and *Escherichia coli* were used to assess the antibacterial activity. The study was performed on the kurarinone isolated from the chloroform fraction of *S. flavescens* methanolic root extract. Kurarinone showed potential antibacterial against *B. subtilis*, *B. cereus*, and *S. aureus* (≈15 zone of inhibition, ZOI). Kurarinone did not show inhibitory potential against *E. coli*. The antibacterial activity was compared with the standard antibacterial agent, Ampicillin (Ryu et al., 2008). In an interesting study, Chong et al. (2013) reported the antibacterial activity of kurarinone nanoparticles deposited onto a filter material. The antimicrobial potential durability and nanoparticles morphology was assessed over a time period of 5 months. The study was designed to tackle the toxicity and infection related problems that arise from airborne biological particles such as bacteria. The ethanolic solution of freeze-dried *S. flavescens* whole plant powder and Gram-positive/negative bacteria (Gram-positive *Staphylococcus epidermidis* and *Escherichia coli*) were used for the study. The bacterial inactivation percentage was calculated by using the (CFU_experiment_/CFU_control_) × 100. CFU_experiment_ and CFU_control_ represented the concentration of bacterial colonies obtained from the test and control groups, respectively. The major components in the ethanolic fraction of the test material were studied, and kurarinone was one of them. The study showed that kurarinone chemical degradation was significantly lower in comparison to other test compounds over the 5-month period. The study established the use of natural product nanoparticles for the antimicrobial potential of filters (Chong et al., 2013).

Recently, Min et al. (2020) reported the anti-human coronavirus infection potential in kurarinone. The human coronavirus-OC43 (HCoV-OC43) infected lung fibroblast cell line (MRC-5) was utilized to study the anti-corona virus infection. The cellular toxicity, quantification of virus RNA copy number, viral protein expression, quantification of cytokine at mRNA level, and other parameters were studied in transfected MRC-5 cells treated with kurarinone. The test compound inhibited the growth of virus infected fibroblast cell line at ≈3.5 µM IC_50_ concentration (Min et al., 2020). The cells were incubated with kurarinone at 3.5 µM for 4 days and then studied for the virus-induced cytopathic effect using light microscopy. The results were compared with the non-treated and remdesivir treated groups. The result revealed promising potential in kurarinone against virus-induced cytopathic effect in transfected fibroblast. qRT-PCR and Western blot analysis revealed that kurarinone significantly reduced the expression of viral proteins at mRNA and protein levels in the transfected cells. Further, the time-of-addition assay showed virus-induced cytopathic effect of kurarinone was significant (in co-administered and post-viral administered treatment) (Min et al., 2020). Impairment in autophagy in host cells is an important phenomenon in virus-induced cytopathic effect. Studies showed that the kurarinone has potential to mitigate the virus-induced autophagy by modulating the expression of LC3-II/LC3-I ratio at 5 µM (Min et al., 2020).

### Antioxidant Activity

Jung et al. (2004) first assessed the antioxidant potential of kurarinone isolated from the ethanolic fraction of the methanol extract of *Albizzia julibrissin* (Leguminosae). The antioxidant potential was assessed using 1,1-diphenyl-2-picrylhydrazyl (DPPH) radical scavenging activity (Jung et al., 2004). Later, Piao et al. (2006) reported *in vitro* radical scavenging potential of kurarinone in biochemical and cell culture experiments. The kurarinone was isolated from aqueous, methylene chloride, and butanol fractions of *S. flavescens* root methanol extract using bioactivity-guided fractionation and isolation approach. The radical scavenging potential was tested using DPPH radical scavenging assay. The protective efficacy of kurarinone against free radical (2,2′-azobis(2-amidinopropane) dihydrochloride or (AAPH)-induced damage in kidney cells (LLC-PK_1_). Kurarinone (butanol fraction) showed potential dose dependent DPPH radical scavenging activity (7.73 µg/ml IC_50_). The butanol fraction produced dose dependent protective efficacy in AAPH induced oxidative damage in LLC-PK_1_ cells. The non-treated and AAPH treated cells were considered as control experimental setup to compare the results. AAPH decreased the LLC-PK_1_ cell viability by 60%. Kurarinone treatment restored the cell viability by 70–90% at 5–50 µg/ml concentration (Piao et al., 2006).

Jeong et al. (2008) studied the copper-induced low-density lipoprotein (LDL) oxidation of *S. flavescens* root isolated kurarinone. The LDL was isolated from human plasma and oxidized with copper to produced oxidized LDL. Conjugate diene formation, malondialdehyde (MDA) estimation, and REM (relative electrophoretic mobility) the assay was performed to assess the anti-oxidative effect of kurarinone in cu-induced LDL oxidation model. Dose and time dependent decreased MDA (IC_50_ 14.5 µM) and diene production (at 5 µM), respectively, were observed in a kurarinone treated experimental group (Jeong et al., 2008). REM assay showed that at higher concentrations (20–80 µM) kurarinone inhibited the formation of oxidized LDL (from wild type non-oxidized LDL). Further, the effect of kurarinone on LDL oxidation was also studied by assessing the fragmentation of lipoprotein apoB-100 using the SDS-PAGE technique. Results showed 56–89% protection against apoB-100 oxidative modification at 40–160 µM concentration in SDS-PAGE based assay. The study revealed the apoB-10 fragmentation protection against LDL oxidation (Jeong et al., 2008). Zhou et al. (2018) assessed the antioxidant potential of kurarinone containing *S. flavescens* in ultrasonic-assisted optimized isolated flavonoid fraction. The HPLC analysis of the fraction revealed the presence of kurarinone as one of the major constituents of *S. flavescens* extract. The fraction was subjected to test the antioxidant potential in terms of DPPH radical and hydroxyl radical scavenging activity. Dose dependent antioxidant activity was observed with 0.984 and 1.084 mg/g IC_50_ in DPPH and OH radical scavenging assay, respectively (Zhou et al., 2018).

### Neuroprotective Efficacy

Jeong et al. (2008) studied the neuroprotective efficacy of flavones isolated from *S. flavescens* in glutamate induced experimental model. The glutamate mediated neurotoxicity (oxidative stress) was induced in immortalized mouse hippocampal cell line (HT22). The heme oxygenase (HO)-1 activity and ROS generation were studied in *S. flavescens* flavones treated HT22 cells. Kurarinone did not show the effect on (HO)-1 activity and ROS generation in the experimental setup (Jeong et al., 2008). Park et al. (2009) studied neuroprotective effects of *S. flavescens* ethyl acetate extract (alkaloid free) by studying its efficacy against focal cerebral ischemia (FCI) in the experimental rat model. HPLC analysis of the solvent-portioned extract (by using various solvents such as water, hexane, etc.) revealed ≈46% kurarinone in the test material. The middle cerebral artery occlusion (MCAO) method was to induce FCI in the Sprague-Dawley rats. The extract showed dose dependent decrease in the sodium nitroprusside induced cell mortality in SH-SY5Y (neuronal) cells. Kurarinone containing test extract pre-treatment (0.2–10 µg/ml) increased the apoptotic population (by modulating the protein expression of caspase-3 and extent of DNA fragmentation) which was decreased in the sodium nitroprusside treated group. After the satisfactory results in the *in vitro* study, the author studied the neuroprotective efficacy of the test sample in the MCAO model. Results showed that kurarinone containing test extract significantly reduced the severity of neurological deficits in the experimental rats (Park et al., 2009).

### Channel and Transporter Activity Modulation

Voltage-gated Ca^+^ channel modulates the concentration of calcium ions into the cells by checking their passage across the cell membrane and thereby affect muscle contraction. Yamahara et al. (1990) studied the muscle relaxation potential of *S. flavescens* root methanolic (MT) and ethanolic (ET) fractions in the thoracic aorta of rabbits and rats. The potassium chloride (50 mM) was used to induce contraction in the test sample and then the relaxation efficacy of *S. flavescens* root fractions was studied. The results were compared with the papaverine (10^−4^ M) induced contraction (considered as 100%). The MT and ET fractions at 25–50 µg/ml concentration exert 40–60% muscle relaxation. After portioning the ET fraction in butanol, water, and ET fractions, the ET fraction showed 100% muscle relaxation. To identify the active ingredient the ET fraction was further sub-fractionated into nine parts. Out of which the second sub-fraction was identified as kurarinone, and showed about 100% muscle relaxation in rabbit and rat aorta (Komatsu et al., 1970; Yamahara et al., 1990).

Sodium-glucose cotransporter (SGLT) is known to absorb/re-absorb the glucose molecules into the cells. The SGLT1 and SGLT2 are responsible for the absorption of dietary glucose and reabsorption of body glucose in the proximal tubule, respectively. The inhibition of the SGLT transporter is an attractive target for type 2 diabetes. Sato et al. (2007) studied the SGLT1 and SGLT2 transporter inhibition potential of *S. flavescens* root extracts. Initially, the methanolic extract was portioned into water and ethyl alcohol. The alcoholic fraction was further sub-fractionated into 10 fractions out of which fraction 5 yielded kurarinone. To study the SGLT inhibitory potential of the isolated compounds, [^14^C]methyl-a-D-glucopyranoside uptake was assessed in hSGLT1 or hSGLT2 expressing monkey kidney derived fibroblast like cells (COS-1). At 50 µM, kurarinone containing fraction showed ≈100% SGLT1 and SGLT2 inhibition efficacy. In a further experiment, kurarinone exerted 50% inhibitory potential against GSLT1 and SGLT2 with IC_50_ of 10.4 and 1.7 µM, respectively (Sato et al., 2007).

Gamma-aminobutyric acid type A (GABA_A_) receptors are involved in neurotransmission inhibition through the influx of calcium ions in response to the binding of *γ* -aminobutyric acid to the receptor. GABA_A_ receptor inhibitors are used to treat different neuronal pathophysiological conditions. Yang et al. (2011) studied the GABA_A_ receptors potentiation of kurarinone in *Xenopus* oocytes. The ethanol extract of *S. flavescens* root was prepared and the micro fractionation was done using HPLC to obtain 22 fractions. The kurarinone was identified in fraction 9 and showed maximum potentiation (100% at 10 µM concentration) of GABA_A_ receptors, which were transiently expressed in stages I-IV *Xenopus* oocytes (Yang et al., 2011).

The BK_Ca_ (large conductance Ca-activated K-channel) channel is involved in the relaxation process of the urinary bladder smooth muscle. In over-reactive bladders, the channel possesses therapeutic target potential to control micturition frequency (Lee et al., 2016). Lee et al. (2018) screened ≈800 natural compounds for their BK_Ca_ potentiation efficacy by using cell-based fluorescence assay in hyperactive mutant BK_Ca_ channel expressing AD-293 cells (derived from human embryonic kidney cells). At 5 µM kurarinone increased the BK_Ca_ channel activity as evident by the increase in fluorescence. The mechanistic study revealed that the kurarinone stabilizes the open conformation of the channel. Moreover, the study also reported that the kurarinone treatment decreases the bladder contraction in rats having over-activated urinary bladders and thereby decreased the micturition frequency (Lee et al., 2018).

In diabetic patients, use of SGLT inhibitors may create a urinary tract infection (UTI) problem as the increased glucose concentration in the urine favors bacterial growth. The bacteria used to attach with the host cells by Type 1 pili (which possess FimH protein). Mashraqi et al. (2021) studied the human SGLT transporter and bacterial FimH protein inhibition potential of natural flavonoids using computer aided drug discovery approach. It has been postulated that the natural compound possessing the inhibitory action against both targets may be used as potential anti-diabetic agents having kisser side effects. In the study kurarinone showed potential binding (−7 kcal/mole) against an SGLT transporter but moderate binding (about −4 kcal/mole) with the FimH protein (Mashraqi et al., 2021).

### Tyrosinase Inhibition Potential

Tyrosinase enzyme is an attractive target for disease associated with local hyperpigmentation as well as in the cosmetic industry for skin whitening. The enzyme modulates the melanin biosynthesis and possesses 1-tyrosine hydroxylase and 1-dopa oxidase activities. Son et al. (2003) prepared *S. flavescens* root methanol extract, and after portioning (using water, dichloro-methane, and ethyl acetate (ET)) the fractions were tested for tyrosinase inhibitory activity. The most potent ET fraction was further fractionated in eight sub-fractions. The kurarinone was identified in one of the sub-fractions and showed potent *in vitro* tyrosinase potential with 1.3 µM IC_50_ concentrations (Son et al., 2003). In a different study, Kim et al. (2003) reported significant dose dependent mushroom tyrosinase inhibitory efficacy (≈100%) in kurarinone at 1–50 µM concentration. Ryu et al. (2008) studied the tyrosinase inhibition potential of kurarinone in the *S. flavescencs* methanol extract isolated compounds. The result showed that kurarinone inhibited (IC_50_ 2.2 µM) l-tyrosine oxidation in a dose dependent manner but did not fully inhibit the tyrosinase enzyme activity. Enzyme kinetics study showed that kurarinone decreased the *Vmax* value with increasing concentration. The mushroom tyrosinase enzyme inhibition assay revealed kurarinone as a noncompetitive inhibitor with 4.1 µM inhibition constant (Ryu et al., 2008).

### Other Pharmacological Potential

The accumulation of triacylglycerol in the body is related to several pathophysiological conditions such as coronary heart disease, obesity, diabetes, and hypertriglyceridemia. Diacylglycerol acyltransferase (DGAT) is a committed step in triacylglycerol synthesis, which makes it a suitable target for the management of the abovementioned disease in patients. Chung et al. (2004) studied the DGAT inhibitory potential of kurarinone. In the spectrophotometric method, kurarinone showed dose dependent DGAT inhibition potential with 10.9 µM IC_50_ in microsomal rat liver fractions. Further, the study on Raji cells showed that kurarinone inhibit free long chain in fatty acid based lipid synthesis at 3–10 µM concentrations (Chung et al., 2004). Protein tyrosine phosphatase 1B is known to inhibit the insulin signaling pathway and thus possess potential as an anti-diabetic therapeutic target. Sasaki et al. (2014) reported that kurarinone inhibited the protein with 41.68 µM IC_50_.

De Naeyer et al. (2004) studied the estrogenic potential of kurarinone. The kurarinone was obtained from the bioactivity-guided sub-fractionation of the *S. flavescens* phenolic extract. The activity was studied in the Ishikawa Var-I bioassay and yeast model. Kurarinone showed potent dose dependent estrogenic activity among the test compounds with 4.6 and 1.6 µM EC_50_ in the yeast screen and Ishikawa Var-I bioassay (De Naeyer et al., 2004).

Glucosidase is an important enzyme related to digestion of carbohydrates, glycoprotein synthesis, and degradation of glycoconjugates. Glucosidase inhibitors are well studied for type 2 diabetes, cancer, and other diseases. Kim et al. (2006) studied the glycosidase inhibition potential of kurarinone isolated from *S. flavescens* root extract and sub-fractionation. The glycosidase activity was assessed for α-glucosidase, β-galactosidase, α/β amylase, and invertase enzymes. Kurarinone showed about 99 and 54% α-glucosidase and β amylase inhibition potential with 45 and 980 µM IC_50_ concentrations, respectively. Further, the enzyme kinetics study showed that kurarinone is a noncompetitive inhibitor of α-glucosidase with 6.8 µM *K*_*i*_ (inhibitory constant) (Kim et al., 2006).

Gao et al. (2007) studied the effect of kurarinone on renal trans-differentiation and interstitial fibrosis in the experimental rat model. The renal interstitial fibrosis rat model was utilized and kurarinone treatment was done at 100 mg/kg body weight. The serum biomarkers (such as creatinine, protein content, albumin, blood urea nitrogen, etc.), pathological markers (in renal tissue), and molecular markers (TGF-β1, αSMA, Smad3, collagen I, etc.) of the disease were studied using appropriate techniques. The kurarinone treated experimental group showed significant down regulated TGF-β1 and collagen I expressions. The study concluded that kurarinone may exert the anti-fibrosis effect through Smad3 down expression (Gao et al., 2007).

Aldose reductase (AR) an NADPH-dependent oxidoreductase converts excess glucose in sorbitol and ultimately in fructose. Later, their accumulation hampers the normal metabolic process and creates complications in diabetic patients. Excess amounts of sorbitol and fructose also produce reactive dicarbonyl species much related to AGE (advanced glycation end products) formation. Jung et al. (2008) reported the AR (rat lens and human recombinant ARs) and AGE inhibition potential in kurarinone using the spectrophotometric method. Kurarinone showed about 31–65% rat lens AR inhibition potential at 0.4–2 µg/ml concentration with 2.99 µM IC_50_. The human recombinant AR inhibition potential was assessed at 1 and 5 µg/ml, which showed 45 and 75% inhibition of the AR with 3.81 µM IC_50_. Kurarinone did not exert the AGE inhibitory efficacy (Jung et al., 2008).

Phytochemicals are known to increase osteoblastic cell proliferation and alkaline phosphate activity both *in vitro* and *in vivo*. Plant product-based management of bone associated diseases such as osteoporosis is a cost-effective strategy. Keeping these facts in mind, Wang et al. (2011) studied the osteogenic effect of flavonoids isolated from *Drynaria fortune*. The study isolated kurarinone from the plant extract which did not show the osteogenic effect at all the test concentrations (10–1,000 nm) in the osteoblastic UMR 106 cells. At higher concentrations, kurarinone increased the ALP activity about 74% (Wang et al., 2011). Xanthine oxidase is an important drug target used for gout, hyperuricemia, ischemic tissue/vascular injuries, inflammation related diseases, and myocardial infarction. Suzuki et al. (2013) studied the xanthine oxidase inhibition potential of kurarinone. The result showed that kurarinone was active against the enzyme only at a higher concentration (100 µM) (Suzuki et al., 2013).

Human carboxylesterases 2 (hCE2) is an important type I xenobiotic metabolism enzyme involved in the ester group metabolism. It detoxifies several environmental toxins in the body as well as hydrolyzes the ester group present in several therapeutic agents. Song et al. (2019) reported the hCE2 inhibitory potential of kurarinone *in vitro*. Kurarinone showed more than 90% hCE2 inhibition potential with 1.46 µM IC_50_ concentration. The activity was dose dependent. Kurarinone was found to be an uncompetitive type inhibitor of hCE2 with 1.73 µM inhibition constant (*K*_*i*_) (Song et al., 2019)*.*

### Bioavailability, Metabolism, and Toxicity

*Sophora flavescens* is a medicinal herb and possesses hepato-protective phytochemicals. Zhixue capsule, a Chinese herbal prescription, encompasses *S. flavescens* extract. The capsule was found to exert dose dependent hepatotoxicity in primary rat hepatocytes (Yu et al., 2013). The *S. flavescens* possess hepato-protective phytochemicals, so how can it show hepato-toxicity? To solve this puzzle, Yu et al. (2013) studied the hepatotoxicity of the *S. flavescens* phytoconstituents in experimental rats. The rats were administered with the *S. flavescens* methanol extract at 1.25 and 2.5 g/kg body weight, twice a day. The treatment was carried out for 3 days. After the treatment, serum alanine transaminase (ALT), AST, and liver histopathology were studied. The result showed an increased concentration of the enzyme markers and damaged liver histology. The extract was sub-fractionated into eight fractions using the semi-preparative HPLC technique. The hepatotoxic effect of kurarinone (subfraction) in rat primary hepatocyte and HL-7702 cells (at 50, 100, and 200 µg/ml concentration) showed significant toxicity (≈30 and 48 µM IC_50_, respectively) (Yu et al., 2013). Jiang et al. (2017) studied the mechanism of kurarinone toxicity in *in vitro* and *in vivo* experimental models. The rats were administered with 1.25 and 2.5 g/kg kurarinone for 14 days. The *in vitro* test in HEK293 cells showed that kurarinone glucuronide get internalized into the cells through OATP1B3 transporter, which is responsible for the entry of therapeutic molecules in the hepatic cells. Inside the hepatocyte, kurarinone inhibits the PPAR-α pathway and reduces L-carnitine which leads to lipid accumulation and liver cell injury (Jiang et al., 2017).

Zhang et al. (2015) developed a method to determine the kurarinone concentration in biological fluids (rat plasma) using UPLC-MS/MS technique. The kurarinone was administered at a dose of 10 mg/kg body weight. The result showed that kurarinone was found for about 1 h in the plasma; after that, the concentration decreased very fast and becomes zero at the sixth hour (Zhang et al., 2015). A similar study was performed by Yang et al. (2016), which administered a higher amount of kurarinone (25 and 500 mg/kg by weight) and the stability in rat plasma was monitored for 12 h (Yang et al., 2016). In a similar but different study, Huang et al. (2020) developed a UPLC-MS/MS based method to detect the kurarinone levels in dog plasma. In this study, kurarinone was administered at 2 and 20 mg/kg body weight, and the blood plasma level was monitored for 25 h (Huang et al., 2020).

*S. flavescens* extract has been reported for its effect on xenobiotic metabolism enzyme modulation. For the first time, Qin et al. (2020) studied the interaction of kurarinone with cytochrome P450 and UDP-glucuronosyl transferase enzymes in the liver microsomes and recombinant human supersomes. The result showed that kurarinone inhibited UDP-glucuronosyl transferase (UGT1A1/A6) and cytochrome P450 (CYP2C9, 1A2, and 2D6) effectively at 100 µM concentrations (Qin et al., 2020). In a different study, Zhang et al. (2016) reported the mode of kurarinone metabolism in human liver microsomes. The study showed that it is metabolized in liver microsomes by the glucuronidation detoxification pathway. The study provided important information about the safe usage of kurarinone as a therapeutic molecule.

### Clinical Study and Patents

In an important clinical study, Pan et al. (2005) studied the combinatorial effect of kurarinone and interferon alpha-1b (IFNa-1b) in chronic hepatitis B patients. The kurarinone and IFNa-1b were added with the conventional hepatitis B treatment to the patients (Group A). Further, Groups B and C represented the addition of kurarinone and IFNa-1b alone with the treatment, respectively. The treatment was carried out for 6 months and during the next 6 months follow up of the patients was carried out to study the disease associated pathological markers such as liver histology, tissue, and serum TGF-β levels, ALT, etc. The result showed that only Group A significantly lowered the liver fibrosis scores and mitigated the pathophysiological markers (Pan et al., 2005). Natural formulation containing kurarinone has been patented for its therapeutic efficacy against hepatitis and cancer (patent number CN1970001B and CN1961898A, respectively). The fact substantiates the therapeutic efficacy of kurarinone.

## Conclusion and Future Prospects

An extensive literature survey on kurarinone (from its first isolation in 1970 to date) revealed the potent therapeutic potential in kurarinone against various disease/ailments. The present review summarizes the chemoprevention and other pharmacological activities of kurarinone (a natural flavanone). It exhibits cell proliferation inhibition, cell cycle arrest, apoptosis induction, anti-metastasis, and stress-induced cytotoxicity in different cancer cells. Kurarinone is an important ingredient of several Chinese medicinal products. The present review activities justify the traditional use of kurarinone in the medicinal system. Although some authors reported hepatotoxicity of kurarinone, but in most of the studies authors showed cancer cell selectivity property in it. Further, a clinical study also showed the non-toxic therapeutic property in kurarinone. Thus, the targeted study should be designed to study the bioavailability and toxicity profile of kurarinone in a pre-clinical and clinical setup. Most of the *in vivo* mechanistic anticancer potential was studied in the lung cancer experimental model, leaving other devastating cancers such as breast, colon, etc. This creates a large thrust area for further research in other cancers at pre-clinical and clinical levels. The literature is silent about the effect of kurarinone on other hallmarks of cancer such as angiogenesis, cancer stemness, etc. Experimental studies showed that kurarinone has the potential to inhibit NF-кB activation directly and indirectly (by lowering TNF-α induction) in disease models such as inflammatory disease (collagen-induced arthritis) and cancer. In arthritis, higher expression of inflammatory molecules (interleukins and TNF-α) activates the NF-кB inflammatory pathway which results in the disease progression. In cancer, NF-кB activation is known to inhibit the apoptotic process which results in cancer cell survival. Thus, the literature indicates that the inhibition of the NF-кB mediated pathway is one of the important mechanisms behind the pharmacological potential of kurarinone. The pharmacological potential of the phytochemical in other diseases that are associated with the NF-кB inflammatory pathway should be studied in appropriate *in vitro* and *in vivo* models. Furthermore, the literature showed that kurarinone can inhibit NF-кB, JAK/STAT, and Akt pathways in different disease experimental models. These pathways are well-known therapeutic targets for cancer and inflammatory diseases. Activation of JAK/STAT, and Akt pathways are well correlated with the tumor initiation, progression, and metastasis. Inflammatory molecules are known to up-regulate the notch signaling pathway which results in various pathological conditions including cancer. Thus, kurarinone might inhibit the notch signaling mediated pathological effects by regulating the NF-кB inhibition mediated inflammatory response. Although the literature showed antioxidant potential in kurarinone, its neutraceutical and/or food supplementation potential has not yet been established. Thus, the *in vitro* and *in vivo* studies regarding neutraceutical/food supplementation potential of the phytochemicals are very much required. The results of the studies might open the transformation of kurarinone from a therapeutic molecule to a potential neutraceutical. Moreover, the metabolism and xenobiotic metabolic enzyme inhibition mediated in-depth efficacy in the associated disease model have not been fully investigated well. Overall, the present study provides a comprehensive update on the therapeutic potential of kurarinone, which may foster the discovery and development of novel therapeutic agents for the treatment of various diseases including cancer.

## Glossary

αSMA: αsmooth muscle actin
AST: aspartate aminotransferase
eIF2a: eukaryotic translation initiation factor 2 α
EGFR: epidermal growth factor receptor
ERK1/2: extracellular signal regulated kinase 1/2
Grp78: glucose-regulated protein
GSH-Px: plasma glutathione peroxidase
Her-2: human epidermal growth factor receptor-2
HEK293: human embryonic kidney 293
H_2_O_2_: hydrogen peroxide
IL-6/8/17A: interleukin-6/8/17A
iNOS: inducible nitric oxide synthase
JAK/STAT: Janus kinase/signal transducer and activator of transcription
JNK: c-Jun N-terminal kinase
KDR: kinase insert domain receptor
KEAP: Kelch-like-ECH-associated-protein
LPS: lipopolysaccharide
Mcl-1: myeloid leukemia and chlamydia
MDA: melanoma differentiation associated protein
MIC: minimum inhibitory concentration
MMP3/2/9: matrix metalloproteinase
NADPH: nicotinamide adenine dinucleotide phosphate
NF-κB: necrosis factor-κB
NO: nitric oxide
Nrf2: nuclear factor erythroid 2-related factor 2
OATP1B3: organic anion transporting polypeptide 1B3
PARP: poly (ADP-ribose) polymerase
PERK-eIF2α: protein kinase R (PRK) like endoplasmic reticulum
PGK: phosphoglycerate kinase
p90RSK: serine/threonine-protein kinase
ROS: reactive oxygen species
SOD: superoxide dismutase
S6RP: S6 ribosomal protein
TGF-β1: Tumor growth factor β1
Th1: type 1T helper
Th17: type 17T helper
TNF-α: tumor necrosis factor-α
TRB3: tribbles homolog
